# Neither Single nor a Combination of Routine Laboratory Parameters can Discriminate between Gram-positive and Gram-negative Bacteremia

**DOI:** 10.1038/srep16008

**Published:** 2015-11-02

**Authors:** Franz Ratzinger, Michel Dedeyan, Matthias Rammerstorfer, Thomas Perkmann, Heinz Burgmann, Athanasios Makristathis, Georg Dorffner, Felix Loetsch, Alexander Blacky, Michael Ramharter

**Affiliations:** 1 Department of Laboratory Medicine, Division of Medical and Chemical Laboratory Diagnostics, Medical University of Vienna, Vienna, Austria; 2 Department of Medicine I, Division of Infectious Diseases and Tropical Medicine, Medical University Vienna, Austria; 3 Department of Laboratory Medicine, Division of Clinical Microbiology, Medical University of Vienna, Vienna, Austria; 4 Section for Artificial Intelligence, Center for Medical Statistics, Informatics and Intelligent Systems, Medical University of Vienna; 5 Department of Infection Control and Hospital Epidemiology, Medical University of Vienna, Währinger Gürtel 18-20 1090, Vienna, Austria; 6 Institute for Tropical Medicine, University of Tübingen, Tübingen, Germany

## Abstract

Adequate early empiric antibiotic therapy is pivotal for the outcome of patients with bloodstream infections. In clinical practice the use of surrogate laboratory parameters is frequently proposed to predict underlying bacterial pathogens; however there is no clear evidence for this assumption. In this study, we investigated the discriminatory capacity of predictive models consisting of routinely available laboratory parameters to predict the presence of Gram-positive or Gram-negative bacteremia. Major machine learning algorithms were screened for their capacity to maximize the area under the receiver operating characteristic curve (ROC-AUC) for discriminating between Gram-positive and Gram-negative cases. Data from 23,765 patients with clinically suspected bacteremia were screened and 1,180 bacteremic patients were included in the study. A relative predominance of Gram-negative bacteremia (54.0%), which was more pronounced in females (59.1%), was observed. The final model achieved 0.675 ROC-AUC resulting in 44.57% sensitivity and 79.75% specificity. Various parameters presented a significant difference between both genders. In gender-specific models, the discriminatory potency was slightly improved. The results of this study do not support the use of surrogate laboratory parameters for predicting classes of causative pathogens. In this patient cohort, gender-specific differences in various laboratory parameters were observed, indicating differences in the host response between genders.

Sepsis is a frequent and complex systemic inflammatory response to a severe infection. The annual incidence is 300 per 100,000 inhabitants^1^ with a mortality reaching up to 50% in certain populations^2^,^3^. The gold standard of laboratory investigations for the diagnosis of sepsis is microbiological confirmation by blood culture analysis. However, blood culture analysis has several drawbacks, including poor cost efficacy due to a low true positive rate in septic patients^4^. Moreover, a median period of three days is necessary to obtain final results from blood culture analysis^4^,^5^.

Early empiric antibiotic therapy using broad spectrum antibiotics is pivotal for patients’ survival^6^,^7^. However the choice of appropriate antimicrobials is complicated by an increase in specific resistance against certain classes of antimicrobial drugs^8^. Early identification of the causative pathogen would therefore enable a better choice of empiric antibiotic therapy.

Clinical and laboratory parameters have been proposed as potential prediction markers for Gram-positive and Gram-negative infections^9^,^10^,^11^,^12^ and are therefore often used, especially by physicians not specialized in clinical infectious diseases to tailor empiric antimicrobial therapy to predicted classes of pathogens. Such parameters involve differential blood cell counts, acute phase proteins and electrolytes^12^,^13^. Although these parameters are essential in patient care, their usefulness for predicting the causative pathogen is not based on firm evidence. The aim of this study was to investigate the diagnostic capacity of single laboratory parameters or their combination to discriminate between patients with Gram-positive and Gram-negative bacteremia.

## Results

### Patient cohort

**Figure 1:**
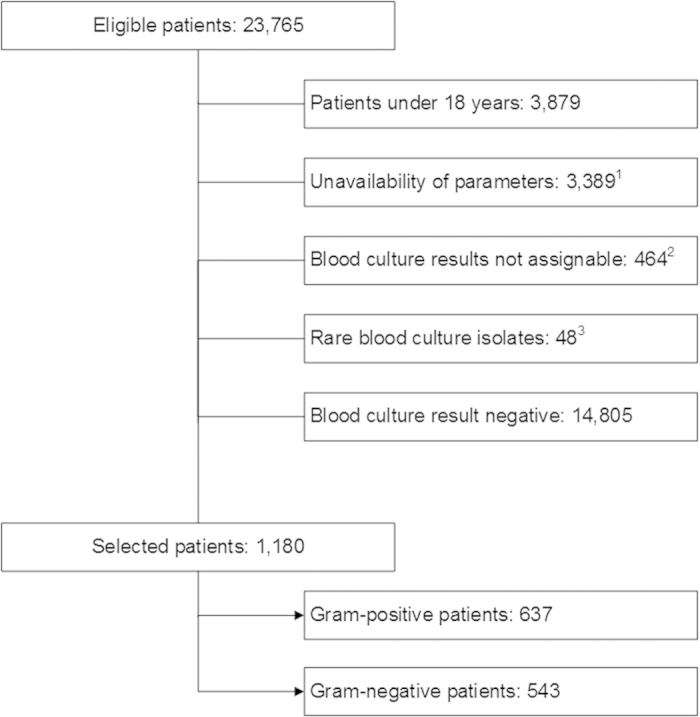
Patient recruitment process. ^1^absence of routine laboratory data for the respective day (more than 95% data missing), ^2^lack of identification of the microorganism at the species level or with potentially contaminated blood, ^3^rarely detected pathogens (less than 0.15% percent).

In total, data from 23,765 patients were screened for inclusion in this study. Patients less than 18 years old (n = 3,879), patients with potentially contaminated blood cultures (n = 464), patients with missing data (n = 3,389) and patients with negative blood culture results (n = 14,805) were excluded from the analysis. To increase the homogeneity of the cohort, patients with rarely detected blood culture isolates (n = 48) were also excluded from further analysis. Finally, data from 1,180 patients were analyzed, including 637 Gram-positive cases (54.0%) and 543 Gram-negative cases (46.0%). Figure 1 presents the patient’s selection process. The pathogens most frequently detected in the Gram-positive group were *Staphylococcus aureus* (n = 259, 40.7%), *Enterococcus faecalis* (n = 76, 11.9%) and *Streptococcus pneumoniae* (n = 64, 10.0%). *Escherichia coli* (n = 333, 61.3%), *Klebsiella pneumoniae* (n = 70, 12.9%) and *Pseudomonas aeruginosa* (n = 69, 12.7%) were the most prevalent Gram-negative pathogens. Within the study population, 58.6% (n = 691) were male and 41.4% (n = 489) were female. The median age was 64 years (25^th^ to 75^th^ percentile: 53–74 years). Female patients suffered relatively less frequently from Gram-positive bacteremia (40.9%) than from Gram-negative bacteremia (59.1%, p = 0.003), whereas this ratio was balanced in male patients (49.6% Gram-positive, 50.4% Gram-negative). Supplementary table S1 presents the distribution of genders in comparison to the Gram-status.

### Single Variable Evaluation

In total, 49 laboratory parameters were evaluated regarding their single predictive power to distinguish between patients with Gram-positives and Gram-negative bacteremia. After applying the Bonferroni-Holm method, significant differences were found in the absolute lymphocyte count, in the relative and absolute count of monocytes, magnesium (all: p < 0.001), phosphate (p = 0.001) and C-reactive protein (CRP, p = 0.001). Details are presented in Table 1. In ROC curve analysis, the best predictive parameters were the absolute and relative amount of monocytes (0.589 ROC-AUC CI: 0.557–0.622; 0.581 ROC-AUC CI: 0.548–0.615 respectively) as well as magnesium (0.573 ROC-AUC CI: 0.538–0.608).

### Multivariable Prediction

**Figure 2:**
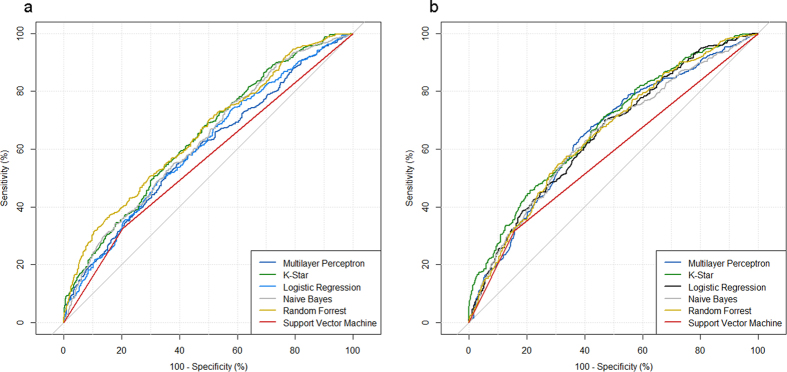
Receiver operating characteristic curve of various models including both genders. (**a**) CFS-selected parameter set, (**b**) wrapper approach-selected parameter set.

The CFS subset evaluator was applied in order to select a relevant parameter set. This feature selection algorithm selected seven parameters, namely gender, amount of lymphocytes (absolute), monocytes (absolute and relative), fibrinogen, creatinine and CRP. For model selection, widely-used machine learning algorithms, including artificial neural networks and support vector machines, were successively applied. Table 2 presents data of the predictive capacities of the machine learning algorithms evaluated and Fig. 2 shows the corresponding ROC-AUC plots. Supplementary Table S2 represents an overview of parameters included in each model. Best performances were shown with the decision tree-based random forest algorithm (RF, ROC-AUC: 0.653, CI: 0.622–0.684) and the K-Star algorithm (ROC-AUC: 0.642, CI: 0.610–0.673). Additionally, a wrapper approach selecting an optimal parameter set for a particular algorithm was applied. Using this approach, the K-Star algorithm with 0.675 ROC-AUC (CI: 0.645–0.705) presented the best performance. This model, consisting of seven parameters, was significantly better (p < 0.001) than the absolute monocyte count (0.589 ROC-AUC), which represented the best predictive individual parameter. The model resulted in a poor calibration (Hosmer-Lemeshow test: p < 0.001), with an inadequate increase in the predicted risk compared to the observed risk (see Supplementary Figure S1a). When applying the Youden index method for cut-off selection, the K-star model resulted in 44.57% sensitivity, 79.75% specificity, 62.79% NPV, and 65.23% PPV for predicting Gram-negative bacteremia.

### Assessment of gender aspect

Since an unequal distribution between Gram-positives and Gram-negative cases and patient’s gender was relatively found, it was speculated that laboratory parameters may present gender-specific differences. Apart from mean corpuscular haemoglobin (MCH) and mean corpuscular volume (MCV), for which gender-specific reference values are established, a significant difference between male and female patients was found in a further four parameters. Females presented with lower blood urea nitrogen (BUN), potassium and creatinine levels, but higher cholesterol levels compared to male patients with bacteremia (all: p < 0.001, see Supplementary Table S3).

Gender-specific data of Gram-positive and Gram-negative cases are shown in Table 3. After applying the Bonferroni-Holm correction, albumin and absolute monocyte count showed significant differences between Gram-positives and Gram-negatives in female patients. Similarly, the distribution of relative and absolute monocyte count, absolute lymphocyte count, CRP, fibrinogen and magnesium significantly differed between male patients with Gram-positive and Gram-negative bacteremia.

**Figure 3:**
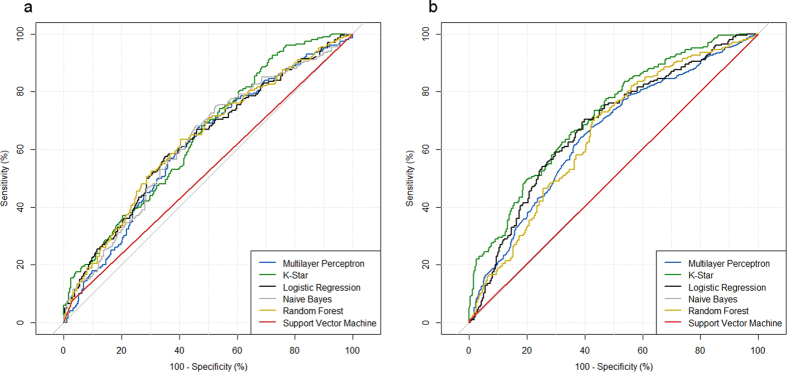
Receiver operating characteristic curve of models including female patients. (**a**) CFS-selected parameter set, (**b**) wrapper approach-selected parameter set.

Since it was evident that patients’ gender had considerable influence on various parameters, gender-specific models were trained. When restricted to female patients, the K-Star algorithm performed best in the parameter subset selected by CFS subset evaluator (0.644 ROC-AUC, CI: 0.595–0.693) as well as in a wrapper approach selected subset (ROC-AUC: 0.716, CI: 0.670–0.761). Figure 3 presents ROC-AUCs of both parameter subsets. When using the Youden Index, the K-star wrapper model reached 65.50% sensitivity, 64.71% specificity, 56.22% PPV and 73.05% NPV for predicting Gram-negatives (see Table 4). The model was not adequately calibrated to the given data (p < 0.001), with an underrating of the risk in low-risk patients and an overrating for high-risk patients (see Supplementary Figure S1b).

**Figure 4:**
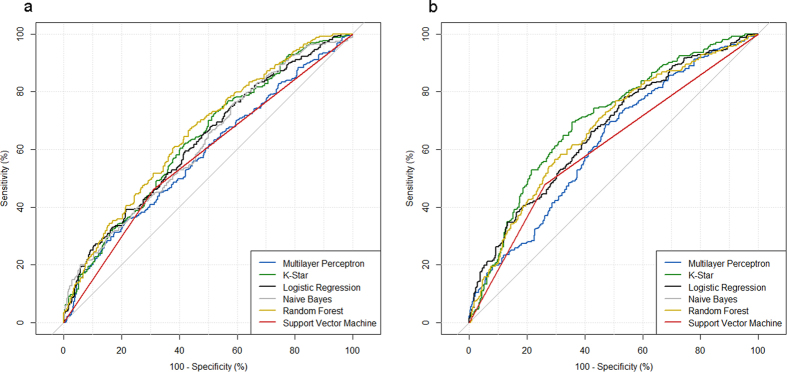
Receiver operating characteristic curve of models including male patients. (**a**) CFS-selected parameter set, (**b**) wrapper approach-selected parameter set.

In male patients, the RF-classifier and the K-star algorithm presented the best ROC-AUCs (RF: 0.657, CI: 0.616–0.700; K-star: 0.633, CI: 0.592–0.674) in the CFS-selected subset. Using the wrapper approach for feature selection, the K-star classifier achieved the best ROC-AUC with 0.699 (CI: 0.660–0.738, see Fig. 4). Male patient-derived model yielded 69.39% sensitivity, 64.37% specificity, 65.75% PPV and 68.09% NPV for the prediction of Gram-negative cases. In contrast to the other models, the calibration curve indicated a better model calibration (see Supplementary Figure S1c). However, especially in the high risk range, a significant deviation between the predicted risk to the observed risk was seen, which was also indicated by the Hosmer-Lemeshow test (p < 0.001).

## Discussion

Improvement of survival in patients with severe bloodstream infections largely depends on the appropriate choice of early empiric antimicrobial therapy. The aim of the present study was to investigate the predictive capacity of highly standardizable parameters to discriminate between Gram-positive and Gram-negative bacteremia.

Among the parameters tested, six variables showed a significant difference between patients with Gram-positive and patients with Gram-negative bacteremia. The absolute monocyte count revealed the highest discriminatory abilities with 0.589 ROC-AUC (CI: 0.557–0.622). In accordance with the literature, CRP was higher in Gram-negative than in Gram-positive infections (p = 0.001), while WBC did not show any significant alterations between Gram-positive and Gram-negative pathogens^12^. Using the K-star algorithm, a model with 0.675 ROC-AUC (CI: 0.645–0.705) was established, resulting in 44.6% sensitivity and 79.8% specificity for detecting Gram-negative bacteremia. Although the model was significantly better than the best single discriminatory parameter (p < 0.001), its ability to estimate the predicted risk was poor. Based on these results, laboratory markers cannot be reliably used to predict classes of bacterial pathogens and this clinical practice therefore should be discouraged. This finding is of particular importance, since the selection of tailored antimicrobial regimens based on unreliable prediction models may lead to a higher risk of treatment failure and ultimately to higher mortality.

Interestingly, a gender-specific aspect in the susceptibility to Gram-negative pathogens as well as in the response to bacteremia in general was noted. As described in the literature, more male than female patients suffered from bacteremia^14^,^15^. However, a significantly lower rate of Gram-positive bacteremias and comparatively higher relative rate of Gram-negative bacteremia was found in females than in male patients (p = 0.003). At this stage it is only possible to speculate as to the reasons for this higher susceptibility of female patients to Gram-negative pathogens. Several potential causes have been postulated, including the influence of hormones, X-chromosomal gene polymorphisms, or expression of cytokines^16^,^17^,^18^,^19^,^20^. Such a gender-specific difference in the cytokine expression was found in several studies, with a more steady expression of pro-inflammatory cytokines in male patients^18^. Furthermore, patients’ gender has been shown to impact on the outcome of patients with severe infections. In several publications, female patients presented a higher mortality rate^21^,^22^,^23^,^24^. In a prospective study conducted at ICUs, the overall mortality was balanced between both genders, but in subgroup analysis restricted to septic patients, females had a significantly higher mortality rate than males (23.1% vs. 13.7%, p = 0.036)^22^.

Moreover, a significant difference between female and male patients was seen in various laboratory parameters without gender-specific reference ranges (see Supplementary Table S3). However, none of these parameters presented predictive capacities for differentiating Gram-positives and Gram-negatives (Tables 2 and 3). When using data aggregated on the basis of gender, the resulting K-star model was slightly superior to models using both genders. However, the predictive capacity of these models as well as their model calibration was poor.

Due to its retrospective design, several limitations must be considered. Firstly, clinical data might have improved the predictive capacities of established models. However, clinical data are difficult to standardize and therefore models incorporating clinical data are difficult to apply in everyday use and their external validity to other health care institutions and settings is questionable. Furthermore, the analysis was restricted to laboratory parameters routinely available during the study period. Non-routinely available parameters, such as lipopolysaccharide binding protein (LBP) and the CD14-ST isoform, have been shown to be associated with Gram-negative sepsis, and might therefore have improved predictive models^11^,^25^. The gender-specific differences in the immunological response of bacteremic patients were unexpected findings in our cohort; however, their effect on the resulting model was limited.

In summary, the results of this study do not support the assumption that surrogate parameters are potential predictors for the classification of causative bacterial pathogens. The usefulness of models consisting of routinely available laboratory parameters to discriminate between Gram-positive and Gram-negative bacteremia was limited. In this study cohort, gender-specific differences in various laboratory parameters were observed, indicating differences in the host response to bacterial blood stream infection.

## Methods

### Study design and data collection

This retrospective cohort study included patients with suspected blood stream infection, treated between January 2006 and December 2010 at Vienna General Hospital. As previously described^26^, all patients for whom their treating physician requested blood culture analysis and a certain panel of laboratory parameters within the same day were screened for eligibility. Those patients with a positive blood culture revealing a bacterial pathogen were included in the study. Exclusion criteria were the patient’s age (less than 18 years), absence of routine laboratory data for the respective day (more than 95% data missing) or negative results from the blood culture analysis. Patients for whom the microorganism could not be identified on the species level or with potentially contaminated blood culture results were excluded from the study. Contaminates were pre-defined according to Hall and Lyman (i.e. coagulase-negative staphylococci, *Corynebacterium spp., Bacillus spp. except Bacillus. anthracis, Proprionibacterium acnes, Streptococcus viridans* and *Clostridiium perfringens*)^27^,^28^. Furthermore, patients with very rarely detected pathogens (less than 0.15% percent) were also excluded from analysis. In total, 49 laboratory parameters, as well as the patient’s age and gender, were statistically analyzed. All laboratory parameters indicated had been analyzed at an ISO 9001:2008 certified facility (Clinical Department of Laboratory Medicine, Medical University Vienna) in accordance to parameter specific standard operating procedures (SOPs).

### Statistical analysis

For statistical analysis, WEKA (Version 3.7.10, GNU General Public License), R (Version 3.0.2, GNU General Public License) and MedCalc (Version 14.8.1, MedCalc Software bvba, Ostend, Belgium) were applied^29^,^30^. Each parameter is characterized as median and interquartile range. For single-variable analysis, Pearson’s chi-squared test, the Mann-Whitney-U test and the area under the receiver operating characteristic curve (ROC-AUC) were used. Due to the high quantity of parameters available, a parameter selection step was necessary to restrict the multivariable analysis to a relevant set of parameters. Parameters were selected using the correlation-feature selection (CFS) subset evaluator and, in parallel, by applying a wrapper approach^31^,^32^. The CFS subset evaluator assesses the predictive power of individual parameters by considering the degree of inter-correlation to the other parameters in the subset. In contrast, the wrapper subset evaluator selects the appropriate parameter set for a particular classification algorithm.

For the establishment of multivariable models, various major classes of machine learning algorithms were employed, including artificial neural networks, support vector machines or Bayes theorem based algorithms. As a reference algorithm, a logistic regression was applied. In brief, (1) the Naïve Bayes classifier (NB) is a simple probabilistic algorithm that assumes that all input parameters are independent from each other and directly applies Bayes’ theorem for classification. (2) The artificial neural network algorithm (ANN) approximates nonlinear functions by superpositions of simple nonlinear basic functions (here, the sigmoid function was used), optimized by a gradient-based algorithm. (3) A support vector machine (SVM) uses a Kernel function to transform the original features into a high-dimensional feature space and finds the linear discrimination with the largest margin (large-margin classifier). In this study, the SMO-SVM algorithm was applied, using normalized attributes and a polynomial kernel. (4) The K-Star algorithm is an instance-based classifier using an entropy distance function. (5) The random forest algorithm (RF) is an ensemble algorithm using decision trees with a bagging approach^33^,^34^,^35^,^36^,^37^,^38^. All algorithms were used in WEKA standard settings. Results were taken from an internal ten-fold cross validation. These were used for ROC-AUC analysis and estimation of the model’s calibration. For this purpose, Hosmer-Lemeshow tests as well as calibration plots were applied^39^. ROC-AUCs were compared by applying the DeLong test as well as the Hanley and McNeil test^40^,^41^,^42^. Cut-off points were set using the Youden index method. Confidence intervals (CI) of binary outcome measures, including sensitivity, specificity, negative predictive value (NPV) or positive predictive value (PPV), were bootstrapped in 2,000 iterations. Statistical significance was defined as p-values less than 0.05. Where appropriate, errors related to multiple testing were corrected by applying the Bonferroni-Holm method.

### Ethical considerations

The study was approved by the local ethics committee of the Medical University of Vienna (EC-number: 333/2011). It was conducted in accordance with the Declaration of Helsinki and the standards for the reporting of diagnostic accuracy studies (STARD). Due to the retrospective study design, informed consent was not required from study participants. During data processing, a consecutive identification number was attributed to the participants in order to assure anonymity.

## Additional Information

**How to cite this article**: Ratzinger, F. *et al.* Neither Single nor a Combination of Routine Laboratory Parameters can Discriminate between Gram-positive and Gram-negative Bacteremia. *Sci. Rep.* **5**, 16008; doi: 10.1038/srep16008 (2015).

## Supplementary Material

Supplementary Information

## Figures and Tables

**Table 1:** Univariate evaluation of available parameters.

No.	Parameter	n	Gram-positive	Gram-negative	p–value^1^	ROC–AUC^2^
1	Age	1180	64.0 (53.0–76.0)	65.0 (53.0–74.3)	ns	ns
2	ALAT (U/L)	1091	30.0 (17.0–61.0)	30.0 (17.8–54.5)	ns	ns
3	Albumin (G/L)	1058	34.7 (29.6–39.0)	31. 5 (26.3–36.7)	ns	ns
4	ALP (U/L)	1065	102.0 (75.0–176.0)	99.00 (71.8–143.5)	ns	ns
5	Amylase (U/L)	886	44.0 (28.0–73.0)	42.50 (30.0–56.3)	ns	ns
6	aPTT (sec)	997	36.2 (33.5–40.6)	39.6 (34.4–43.6)	ns	ns
7	ASAT (U/L)	1075	33.0 (22.0–64.0)	36.0 (26.0–62.0)	ns	ns
8	Basophiles %	1115	0.1 (0.1–0.2)	0.1 (0.1–0.2)	ns	ns
9	Basophiles (G/L)	1155	0.00 (0.00–0.00)	0.00 (0.00–0.00)	ns	ns
10	Bilirubin (mg/dl)	1054	0.9 (0.6–1.8)	1.1 (0.8–1.6)	ns	ns
11	BUN (mg/dl)	1177	19.7 (13.1–30.3)	25.20 (16.3–43.6)	ns	ns
12	Calcium (mmol/L)	1112	2.24 (2.13–2.36)	2.23 (2.13–2.33)	ns	ns
13	CHE (kU/L)	970	4.6 (3.2–6.0)	3.7 (2.6–5.0)	ns	ns
14	Cholesterol (mg/dl)	761	147.0 (114.0–185.0)	127.0 (104.8–165.0)	ns	ns
15	CK (U/L)	994	66.0 (34.0–128.0)	71.0 (31.8–175.3)	ns	ns
16	Creatinine (mg/dl)	1173	1.20 (0.87–1.56)	1.26 (0.87–1.80)	ns	ns
17	CRP (mg/dl)	1176	10.1 (4.5–18.6)	17.0 (7.5–25.6)	0.001	0.558 (0.525–0.591)
18	Eosinophil %	1109	0.2 (0.0–0.7)	0.100 (0.0–0.6)	ns	ns
19	Eosinophils (G/L)	1150	0.0 (0.0–0.1)	0.0 (0.0–0.1)	ns	ns
20	Fibrinogen (mg/dl)	951	535.0 (414.0–673.0)	615.0 (467.3–791.0)	ns	ns
21	GGT (G/L)	1069	71.0 (29.0–171.0)	63.0 (32.8–176.5)	ns	ns
22	Glucoses (mg/dl)	961	124.0 (103.0–152.0)	126 (100.0–164.0)	ns	ns
23	Haematocrit (%)	1175	34.6 (30.3–38.3)	34.4 (29.8–38.1)	ns	ns
24	Haemoglobin (G/L)	1175	11.5 (10.1–12.8)	11.4 (9.6–12.9)	ns	ns
25	LDH (U/L)	1020	235.0 (183.0–297.0)	273.0 (210.85–366.3)	ns	ns
26	Lipases (U/L)	903	21.0 (13.0–34.0)	19.0 (12.0–32.0)	ns	ns
27	Lymphocytes (%)	1093	6.8 (4.5–12.2)	6.2 (3.9–9.9)	ns	ns
28	Lymphocytes (G/L)	1133	0.7 (0.4–1.1)	0.8 (0.5–1.1)	<0.001	0.570 (0.536–0.603)
29	MCH (fl)	1175	29.7 (28.4–30.9)	29.9 (28.3–31.3)	ns	ns
30	MCHC (g/dl)	1175	33.6 (32.8–34.3)	33.45 (32.68–34.53)	ns	ns
31	MCV (pg)	1175	88.5 (85.8–92.4)	88.9 (84.9–94.1)	ns	ns
32	MG (mmol/L)	1043	0.74 (0.65–0.83)	0.78 (0.71–0.89)	<0.001	0.573 (0.538–0.608)
33	Monocytes %	1094	5.4 (3.0–8.3)	6.3 (3.9–8.8)	<0.001	0.581 (0.548–0.615)
34	Monocytes (G/L)	1131	0.6 (0.2–1.0)	0.7 (0.4–1.1)	<0.001	0.589 (0.557–0.622)
35	MPV (fl)	1072	10.1 (9.5–11.0)	10.2 (9.7–11.2)	ns	ns
36	Neutrophiles %	1089	86.5 (78.6–90.6)	86.3 (81.1–90.6)	ns	ns
37	Neutrophiles (G/L)	1089	8.9 (5.6–13.0)	10.20 (6.98–15.03)	ns	ns
38	Normotest (%)	991	81.0 (66.0–95.0)	80.0 (61.0–94.3)	ns	ns
39	PAMY (U/L)	667	19.0 (13.0–31.0)	20.0 (12.0–28.0)	ns	ns
40	PDW (%)	1031	11.8 (10.5–13.5)	12.0 (10.7–13.7)	ns	ns
41	Phosphate (mmol/L)	1087	0.9 (0.7–1.1)	1.0 (0.8–1.2)	0.001	0.561 (0.527–0.595)
42	PLT (G/L)	1174	196.0 (142.0–259.0)	192.0 (129.0–262.5)	ns	ns
43	Potassium (mmol/L)	1064	3.9 (3.5–4.3)	4.0 (3.6–4.3)	ns	ns
44	RBC (T/L)	1132	3.9 (3.5–4.3)	3.80 (3.4–4.3)	ns	ns
45	RDW (%)	1175	14.4 (13.6–15.9)	15.0 ( 13.7–16.4)	ns	ns
46	Sodium (mmol/L)	1139	136.0 (133.0– 139.0)	135 (133.0– 139.0)	ns	ns
47	TP (G/L)	1068	67.0 (58.9–72.1)	66.7 (58.2–74.7)	ns	ns
48	Triglyceride (mg/dl)	761	119.0 (84.0–161.0)	120.0 (84.0–166.0)	ns	ns
49	Uric acid (mg/dl)	964	5.5 (3.7–7.2)	5.65 (3.9–8.3)	ns	ns
50	WBC (G/L)	1132	10.5 (7.1–15.1)	12.1 (8.4–17.4)	ns	ns

Data given as median with interquartile range (Q1, Q3), ^1^Mann Whitney U-test, ^2^area under the receiver operating characteristic curve, ns = not significant. ALAT = alanine aminotransferase, ALP = alkaline phosphatase, aPTT = activated partial thromboplastin time, ASAT = aspartate aminotransferase, BUN = blood urea nitrogen, CHE = cholinesterase, CK = creatinine kinases, CRP = C-reactive protein, GGT = gamma-glutamyl transpeptidase, LDH = lactate dehydrogenase, MCH = mean corpuscular haemoglobin, MCV = mean corpuscular volume, MG = magnesium, MCHC = Mean corpuscular haemoglobin concentration, MPV = mean platelet volume, RBC = red blood cell count, PAMY = pancreas amylase, PDW = platelet distribution width, PLT = platelet count, RDW = red blood cell distribution width, TP = total protein, WBC = white blood cell count.

**Table 2:** Predictive capacities of various machine learning algorithms.

Classifier	All		Female		Male	
	CFS^1^	Wrapper approach	CFS^1^	Wrapper approach	CFS^1^	Wrapper approach
LogReg^2^	0.607 (0.575–0.639)^8^	0.652 (0.621–0.683)^9^	0.627 (0.577–0.677)^22^	0.677 (0.629–0.725)^23^	0.627 (0.586–0.668)^15^	0.664 (0.624–0.704)^16^
NB^3^	0.628 (0.596–0.659)^8^	0.643 (0.611–0.674)^10^	0.623 (0.573–0.674)^22^	0.652 (0.603–0.700)^24^	0.620 (0.579–0.661)^15^	0.669 (0.629–0.710)^17^
ANN^4^	0.598 (0.566–0.631)^8^	0.651 (0.620–0.682)^11^	0.614 (0.563–0.664)^22^	0.632 (0.582–0.682)^25^	0.582 (0.540–0.624)^15^	0.620 (0.578–0.661)^18^
SVM^5^	0.561 (0.536–0.586)^8^	0.581 (0.556–0.605)^12^	0.524 (0.503–0.544)^22^	0.503 (0.498–0.507)^26^	0.575 (0.539–0.611)^15^	0.608 (0.573–0.644)^19^
K–Star^6^	0.642 (0.610–0.673)^8^	0.675 (0.645–0.705)^13^	0.644 (0.595–0.693)^22^	0.716 (0.670–0.761)^27^	0.633 (0.592–0.674)^15^	0.699 (0.660–0.738)^20^
RF^7^	0.653 (0.622–0.684)^8^	0.654 (0.623–0.685)^14^	0.632 (0.582–0.682)^22^	0.707 (0.660–0.754)^28^	0.657 (0.616–0.700)^15^	0.661 (0.621–0.701)^21^

Data is given as ROC-AUC with confidence intervals assessed using bootstrapping (n = 2000 iterations); ^1^correlation-feature selection, ^2^logistic regression, ^3^naive Bayes algorithm, ^4^artificial neural network, ^5^support vector machine ^6^K-Star algorithm, ^7^random forest algorithm, ^8^n = 7 [sex, 16, 17, 20, 28, 34, 35]*(number in brackets indicts parameter number displayed in Table 1), ^9^n = 17 [sex, 1, 3, 10, 12, 13, 17, 19, 25, 26, 28, 32, 34, 43, 44, 46, 47], ^10^n = 13 [sex, 1, 10, 13, 17, 19, 24, 31, 33, 37, 38, 43, 47], ^11^n = 7 [sex, 3, 8, 17, 29, 31, 34], ^12^n = 15 [3, 9, 13, 15, 17, 18, 20, 21, 26, 27, 28, 32, 34, 41, 47], ^13^n = 7 [6, 9, 10, 27, 33, 34, 42], ^14^n = 5 [1, 9, 10, 33, 44], ^15^n = 5 [17, 20, 33, 34, 36], ^16^n = 15 [1, 5, 10, 13, 19, 20, 22, 25, 30, 34, 37, 41, 43, 47, 50], ^17^n = 12 [1, 10, 13, 16, 17, 22, 31, 33, 34, 37, 38, 47], ^18^n = 6 [1, 9, 17, 20, 34, 37], ^19^n = 10 [5, 7, 8, 10, 20, 25, 36, 38, 41, 43], ^20^n = 4 [9, 20, 28, 33], ^21^n = 18 [1, 4, 8, 9, 10, 12, 16, 17, 19, 22, 28, 31, 33, 35, 39, 42, 44, 46], ^22^n = 4 [28, 34, 37, 45], ^23^n = 12 [3, 4, 10, 20, 31, 34, 36, 37, 45, 47, 48, 50], ^24^n = 11 [4, 9, 12, 16, 17, 20, 28, 31, 33, 37, 50], ^25^n = 8 [4, 9, 16, 34, 37, 45, 47, 48], ^26^n = 1[9], ^27^n = 6 [32, 37, 39, 44, 45, 50], ^28^n = 7 [7, 16, 19, 31, 34, 41, 45].

**Table 3:** Gender specific data of parameters with predictive capacities for discriminating Gram-positives and Gram-negatives.

	Female				Male			
	Gram-positive	Gram-negative	p–value^1^	ROC–AUC^2^	Gram-positive	Gram-negative	p–value^1^	ROC–AUC^2^
Albumin (G/L)	35.0 (31.8–39.6)	31.2 (25.4–36.8)	<0.001	0.588 (0.534–0.642)	31.8 (28.0–38.3)	31.5 (27.1–36.8)	ns	ns
CRP (mg/dl)	10.6 (5.6–20.3)	11.8 (5.2–29.2)	ns	Ns	9.70 (3.9–16.3)	17.8 (8.3–23.9)	<0.001	0.583 (0.541–0.626)
Fibrinogen (mg/dl)	607.0 (472.0–717.0)	551.0 (422.5–727.0)	ns	Ns	485.5 (363.8–622.0)	621.0 (488.0–824.0)	<0.001	0.604 (0.557–0.650)
Lymphocytes (G/L)	0.7 (0.4–10.1)	0.9 (0.5–10.3)	ns	Ns	0.7 (0.4–10.3)	0.8 (0.5–10.1)	<0.001	0.584 (0.541–0.626)
MG (mmol/L)	0.76 (0.65–0.85)	0.79 (0.71–0.88)	ns	Ns	0.73 (0.66–0.80)	0.78 (0.71–0.91)	<0.001	0.581 (0.535–0.626)
Monocytes %	5.6 (3.8–8.1)	6.4 (4.2–8.1)	ns	Ns	5.2 (2.7–8.5)	6.2 (3.7–9.4)	<0.001	0.597 (0.553–0.641)
Monocytes (G/L)	0.7 (0.3–1.0)	0.7 (0.4–1.2)	<0.0001	0.591 (0.539–0.644)	0.5 (0.2–1.0)	0.7 (0.5–1.1)	<0.001	0.596 (0.553–0.639)

^1^Mann Whitney U-test, ^2^area under the receiver operating characteristic curve, ns = not significant. CRP = C-reactive protein, MG = magnesium.

**Table 4:** Predictive capacities of K-Star models.

	All	Female	Male
ROC–AUC^1^	0.675 (0.645–0.705)	0.716 (0.670–0.761)	0.699 (0.660–0.738)
Sensitivity^2^	44.57% (40.33%–48.86%)	65.50% (58.47%–72.06%)	69.39% (64.21%–74.22%)
Specificity^2^	79.75% (76.41%–82.80%)	64.71% (58.89%–70.21%)	64.37% (59.09%–69.40%)
Positive Likelihood Ratio^2^	2.20 (1.84–2.64)	1.86 (1.54–2.23)	1.95 (1.66–2.28)
Negative Likelihood Ratio^2^	0.70 (0.64–0.76)	0.53 (0.43–0.66)	0.48 (0.40–0.57)
Positive Predictive Value^2^	65.23% (60.14%–70.07%)	56.22% (49.59%–62.69%)	65.75% (60.61%–70.63%)
Negative Predictive Value^2^	62.79% (59.36%–66.13%)	73.05% (67.17%–78.38%)	68.09% (62.75%–73.09%)

^1^Area under the receiver operating characteristic curve, ^2^for prediction of Gram-negative bacteremia, bootstrapped confidence intervals are given in brackets.
