# Supplementary material for: Neither Single nor a Combination of Routine Laboratory Parameters can Discriminate between Gram-positive and Gram-negative Bacteremia
Source: Sci Rep. 2015 Nov 2;5:16008. doi: 10.1038/srep16008 (PMC4629184; doi:10.1038/srep16008)
Supplement: Supplementary Information [file srep16008-s1.doc]

Supplementary Data

**Neither Single nor a Combination of Routine Laboratory Parameters can Discriminate between Gram-positive and Gram-negative Bacteremia**

Franz Ratzinger^1^, Michel Dedeyan^2^, Matthias Rammerstorfer^2^, Thomas Perkmann^1^, Heinz Burgmann^2^, Athanasios Makristathis^3^, Georg Dorffner^4^, Felix Loetsch^2^, Alexander Blacky^5^, and Michael Ramharter^2,6*^

1 Department of Laboratory Medicine, Division of Medical and Chemical Laboratory Diagnostics, Medical University of Vienna, Vienna, Austria

2 Department of Medicine I, Division of Infectious Diseases and Tropical Medicine, Medical University Vienna, Austria

3 Department of Laboratory Medicine, Division of Clinical Microbiology, Medical University of Vienna, Vienna, Austria

4 Section for Artificial Intelligence, Center for Medical Statistics, Informatics and Intelligent Systems, Medical University of Vienna

5 Clinical Institute for Hospital Hygiene; Medical University of Vienna, Vienna, Austria

6 Institute for Tropical Medicine, University of Tübingen, Tübingen, Germany

| 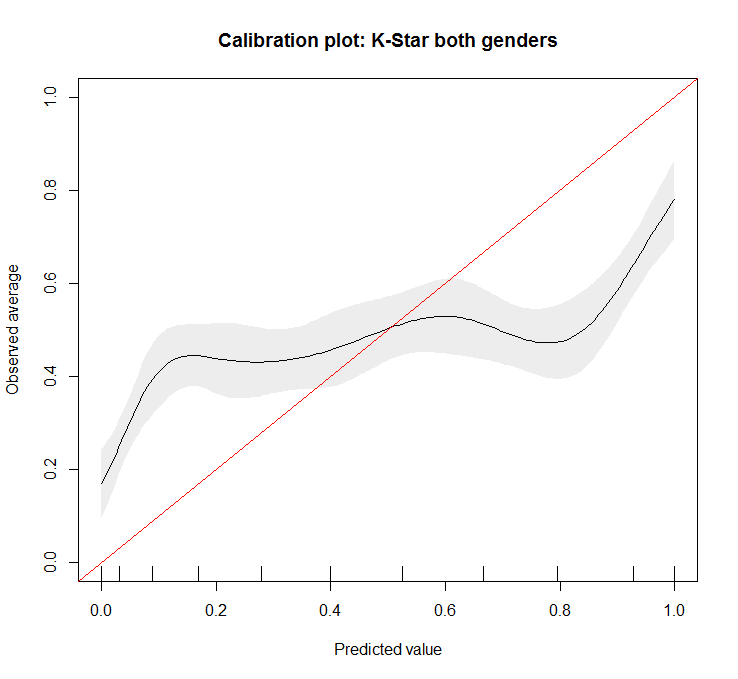**a** | **Supplementary Figure S1:** **Calibration curves of K-Star models** with data of (**a**) both genders, (**b**) females, (**c**) males, grey = standard deviation of the calibration curve computed using the gbm package ^42^. |
| --- | --- |
| 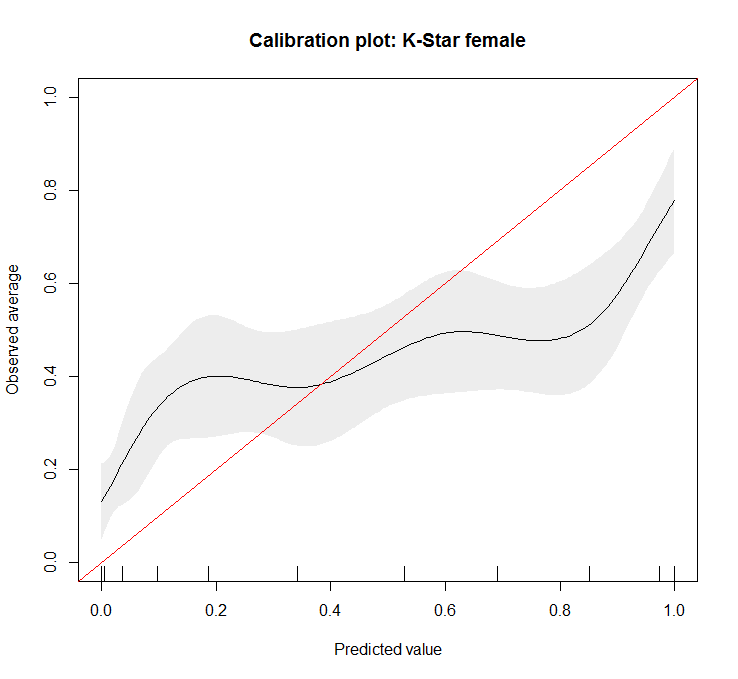**b** |
| 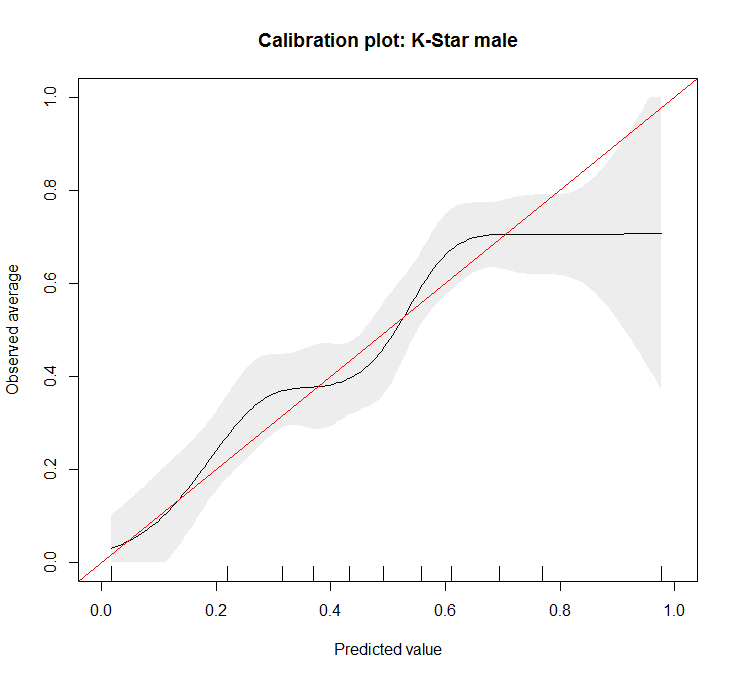**c** |

**Supplementary Table S1:** **Contingency table of the gender distribution in relation to the Gram status**

|  |  | Gram neg | Gram pos | Total |
| --- | --- | --- | --- | --- |
| male | n | 348 | 343 | 691 (58.6%) |
|  | % in sex | 50.4% | 49.6% |  |
| female | n | 289 | 200 | 489 (41.4%) |
|  | % in sex | 59.1% | 40.9% |  |
| Total |  | 637 | 543 | 1180 |

**Supplementary Table S2: Overview of parameters included in each model**

|  |  | ALL | | | | | | | Female | | | | | | | Male | | | | | | |  |  |
| --- | --- | --- | --- | --- | --- | --- | --- | --- | --- | --- | --- | --- | --- | --- | --- | --- | --- | --- | --- | --- | --- | --- | --- | --- |
| # | Parameter | CFS:All | Wrapper:Log | Wrapper:NB | Wrapper:ANN | Wrapper:SVM | Wrapper:K-Star | Wrapper:RF | CFS:All | Wrapper:Log | Wrapper:NB | Wrapper:ANN | Wrapper:SVM | Wrapper:K-Star | Wrapper:RF | CFS:All | Wrapper:Log | Wrapper:NB | Wrapper:ANN | Wrapper:SVM | Wrapper:K-Star | Wrapper:RF |  |  |
| N |  | 7 | 17 | 13 | 7 | 15 | 7 | 5 | 4 | 12 | 11 | 8 | 1 | 6 | 7 | 5 | 15 | 12 | 6 | 10 | 4 | 18 |  | |
| 0 | Sex | X | X | X | X |  |  |  | not applied | | | | | | | | | | | | | |  | |
| 1 | Age |  | X | X |  |  |  | X |  |  |  |  |  |  |  |  | X | X | X |  |  | X |  | |
| 2 | ALAT (U/L) |  |  |  |  |  |  |  |  |  |  |  |  |  |  |  |  |  |  |  |  |  |  | |
| 3 | Albumin (G/L) |  | X |  | X | X |  |  |  | X |  |  |  |  |  |  |  |  |  |  |  |  |  | |
| 4 | ALP (U/L) |  |  |  |  |  |  |  |  | X | X | X |  |  |  |  |  |  |  |  |  | X |  | |
| 5 | Amylase (U/L) |  |  |  |  |  |  |  |  |  |  |  |  |  |  |  | X |  |  | X |  |  |  | |
| 6 | aPTT (sec) |  |  |  |  |  | X |  |  |  |  |  |  |  |  |  |  |  |  |  |  |  |  | |
| 7 | ASAT (U/L) |  |  |  |  |  |  |  |  |  |  |  |  |  | X |  |  |  |  | X |  |  |  | |
| 8 | Basophiles % |  |  |  | X |  |  |  |  |  |  |  |  |  |  |  |  |  |  | X |  | X |  | |
| 9 | Basophiles (G/L) |  |  |  |  | X | X | X |  |  | X | X | X |  |  |  |  |  | X |  | X | X |  | |
| 10 | Bilirubin (mg/dl) |  | X | X |  |  | X | X |  | X |  |  |  |  |  |  | X | X |  | X |  | X |  | |
| 11 | BUN (mg/dl) |  |  |  |  |  |  |  |  |  |  |  |  |  |  |  |  |  |  |  |  |  |  | |
| 12 | Calcium (mmol/L) |  | X |  |  |  |  |  |  |  | X |  |  |  |  |  |  |  |  |  |  | X |  | |
| 13 | CHE (kU/L) |  | X | X |  | X |  |  |  |  |  |  |  |  |  |  | X | X |  |  |  |  |  | |
| 14 | Cholesterol (mg/dl) |  |  |  |  |  |  |  |  |  |  |  |  |  |  |  |  |  |  |  |  |  |  | |
| 15 | CK (U/L) |  |  |  |  | X |  |  |  |  |  |  |  |  |  |  |  |  |  |  |  |  |  | |
| 16 | Creatinine (mg/dl) | X |  |  |  |  |  |  |  |  | X | X |  |  | X |  |  | X |  |  |  | X |  | |
| 17 | CRP (mg/dl) | X | X | X | X | X |  |  |  |  | X |  |  |  |  | X |  | X | X |  |  | X |  | |
| 18 | Eosinophils % |  |  |  |  | X |  |  |  |  |  |  |  |  |  |  |  |  |  |  |  |  |  | |
| 19 | Eosinophils (G/L) |  | X | X |  |  |  |  |  |  |  |  |  |  | X |  | X |  |  |  |  | X |  | |
| 20 | Fibrinogen (mg/dl) | X |  |  |  | X |  |  |  | X | X |  |  |  |  | X | X |  | X | X | X |  |  | |
| 21 | GGT (G/L) |  |  |  |  | X |  |  |  |  |  |  |  |  |  |  |  |  |  |  |  |  |  | |
| 22 | Glucoses (mg/dl) |  |  |  |  |  |  |  |  |  |  |  |  |  |  |  | X | X |  |  |  | X |  | |
| 23 | Haematocrit (%) |  |  |  |  |  |  |  |  |  |  |  |  |  |  |  |  |  |  |  |  |  |  | |
| 24 | Haemoglobin(G/L) |  |  | X |  |  |  |  |  |  |  |  |  |  |  |  |  |  |  |  |  |  |  | |
| 25 | LDH (U/L) |  | X |  |  |  |  |  |  |  |  |  |  |  |  |  | X |  |  | X |  |  |  | |
| 26 | Lipases (U/L) |  | X |  |  | X |  |  |  |  |  |  |  |  |  |  |  |  |  |  |  |  |  | |
| 27 | Lymphocytes (%) |  |  |  |  | X | X |  |  |  |  |  |  |  |  |  |  |  |  |  |  |  |  | |
| 28 | Lymphocytes (G/L) | X | X |  |  | X |  |  | X |  | X |  |  |  |  |  |  |  |  |  | X | X |  | |
| 29 | MCH (fl) |  |  |  | X |  |  |  |  |  |  |  |  |  |  |  |  |  |  |  |  |  |  | |
| 30 | MCHC (g/dl) |  |  |  |  |  |  |  |  |  |  |  |  |  |  |  | X |  |  |  |  |  |  | |
| 31 | MCV (pg) |  |  | X | X |  |  |  |  | X | X |  |  |  | X |  |  | X |  |  |  | X |  | |
| 32 | MG (mmol/L) |  | X |  |  | X |  |  |  |  |  |  |  | X |  |  |  |  |  |  |  |  |  | |
| 33 | Monocytes % |  |  | X |  |  | X | X |  |  | X |  |  |  |  | X |  | X |  |  | X | X |  | |
| 34 | Monocytes (G/L) | X | X |  | X | X | X |  | X | X |  | X |  |  | X | X | X | X | X |  |  |  |  | |
| 35 | MPV (fl) | X |  |  |  |  |  |  |  |  |  |  |  |  |  |  |  |  |  |  |  | X |  | |
| 36 | Neutrophiles % |  |  |  |  |  |  |  |  | X |  |  |  |  |  | X |  |  |  | X |  |  |  | |
| 37 | Neutrophiles (G/L) |  |  | X |  |  |  |  | X | X | X | X |  | X |  |  | X | X | X |  |  |  |  | |
| 38 | Normotest (%) |  |  | X |  |  |  |  |  |  |  |  |  |  |  |  |  | X |  | X |  |  |  | |
| 39 | PAMY (U/L) |  |  |  |  |  |  |  |  |  |  |  |  | X |  |  |  |  |  |  |  | X |  | |
| 40 | PDW (%) |  |  |  |  |  |  |  |  |  |  |  |  |  |  |  |  |  |  |  |  |  |  | |
| 41 | Phosphate(mmol/L) |  |  |  |  | X |  |  |  |  |  |  |  |  | X |  | X |  |  | X |  |  |  | |
| 42 | PLT (G/L) |  |  |  |  |  | X |  |  |  |  |  |  |  |  |  |  |  |  |  |  | X |  | |
| 43 | Potassium (mmol/L) |  | X | X |  |  |  |  |  |  |  |  |  |  |  |  | X |  |  | X |  |  |  | |
| 44 | RBC (T/L) |  | X |  |  |  |  | X |  |  |  |  |  | X |  |  |  |  |  |  |  | X |  | |
| 45 | RDW (%) |  |  |  |  |  |  |  | X | X |  | X |  | X | X |  |  |  |  |  |  |  |  | |
| 46 | Sodium (mmol/L) |  | X |  |  |  |  |  |  |  |  |  |  |  |  |  |  |  |  |  |  | X |  | |
| 47 | TP (G/L) |  | X | X |  | X |  |  |  | X |  | X |  |  |  |  | X | X |  |  |  |  |  | |
| 48 | Triglyceride (mg/dl) |  |  |  |  |  |  |  |  | X |  | X |  |  |  |  |  |  |  |  |  |  |  | |
| 49 | Uric acid (mg/dl) |  |  |  |  |  |  |  |  |  |  |  |  |  |  |  |  |  |  |  |  |  |  | |
| 50 | WBC (G/L) |  |  |  |  |  |  |  |  | X | X |  |  | X |  |  | X |  |  |  |  |  |  | |

ALAT = alanine aminotransferas**e**, ALP = alkaline phosphatase, aPTT = activated partial thromboplastin time, ASAT = aspartate aminotransferase, BUN = blood urea nitrogen, CHE = cholinesterase, CK = creatinine kinases, CRP = C-reactive protein, GGT = gamma-glutamyl transpeptidase, LDH = lactate dehydrogenase, MCH = mean corpuscular haemoglobin, MCV = mean corpuscular volume, MG = magnesium, MCHC = Mean corpuscular haemoglobin concentration, MPV = mean platelet volume, RBC = red blood cell count, PAMY = pancreas amylase, PDW = platelet distribution width, PLT = platelet count, RDW = red blood cell distribution width, TP = total protein, WBC = white blood cell count.

**Supplementary Table S3: Gender differences in patients with bacteremia**, restricted to parameters with a statistically significant difference after application of the Bonferroni-Holm correction.

|  | Female patients | Male patients | p–value^1^ |
| --- | --- | --- | --- |
| BUN (mg/dl) | 20.1 (13.4–37.1) | 24.6 (15.9–41.3) | <0.001 |
| Cholesterol (mg/dl) | 141 (111.5–185.0) | 126 (96.8–161.3) | <0.001 |
| Creatinine (mg/dl) | 1.10 (0.79–1.63) | 1.29 (0.95–1.89) | <0.001 |
| MCH (fl) | 29.5 (28.1–30.9) | 30.1 (28.5– 31.4) | <0.001 |
| MCV (pg) | 88.0 (84.0–91.4) | 89.3 (85.2–93.3) | <0.001 |
| Potassium (mmol/L) | 3.88 (3.50–4.26) | 4.08 (3.7–4.47) | <0.001 |

BUN = blood urea nitrogen, MCH = mean corpuscular haemoglobin, MCV = mean corpuscular volume, ^1^Mann Whitney U-test.
